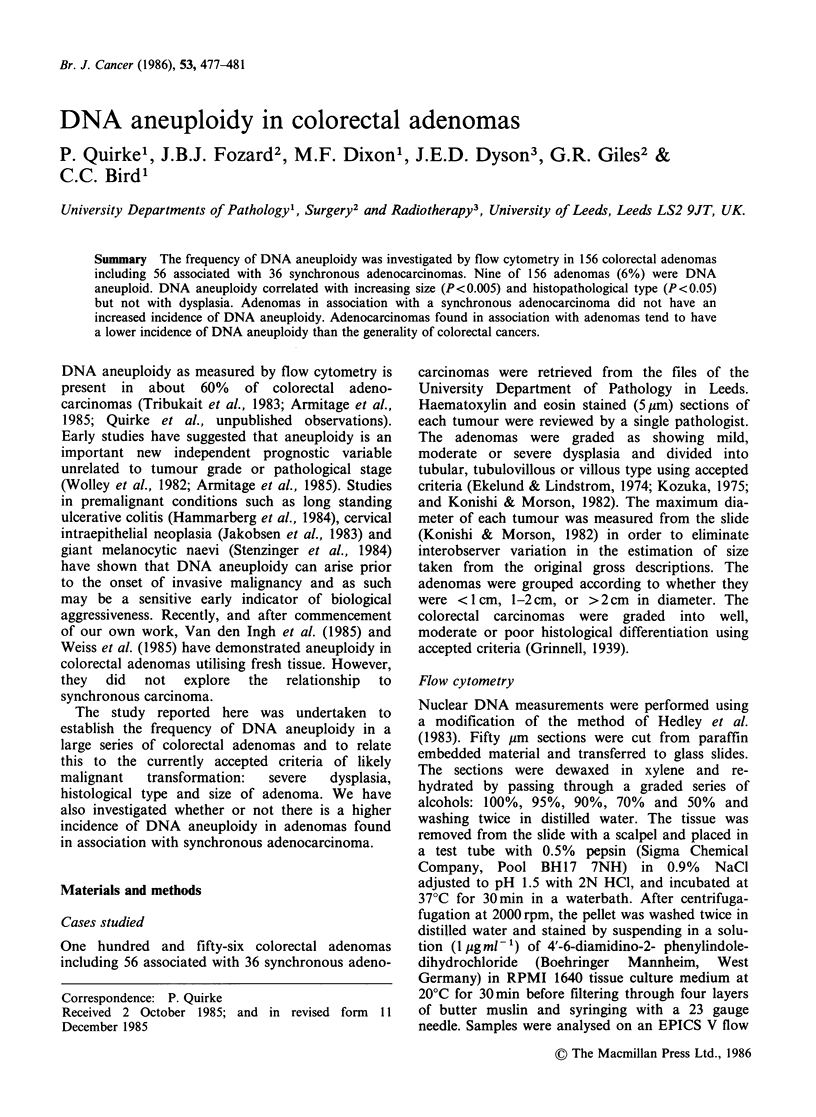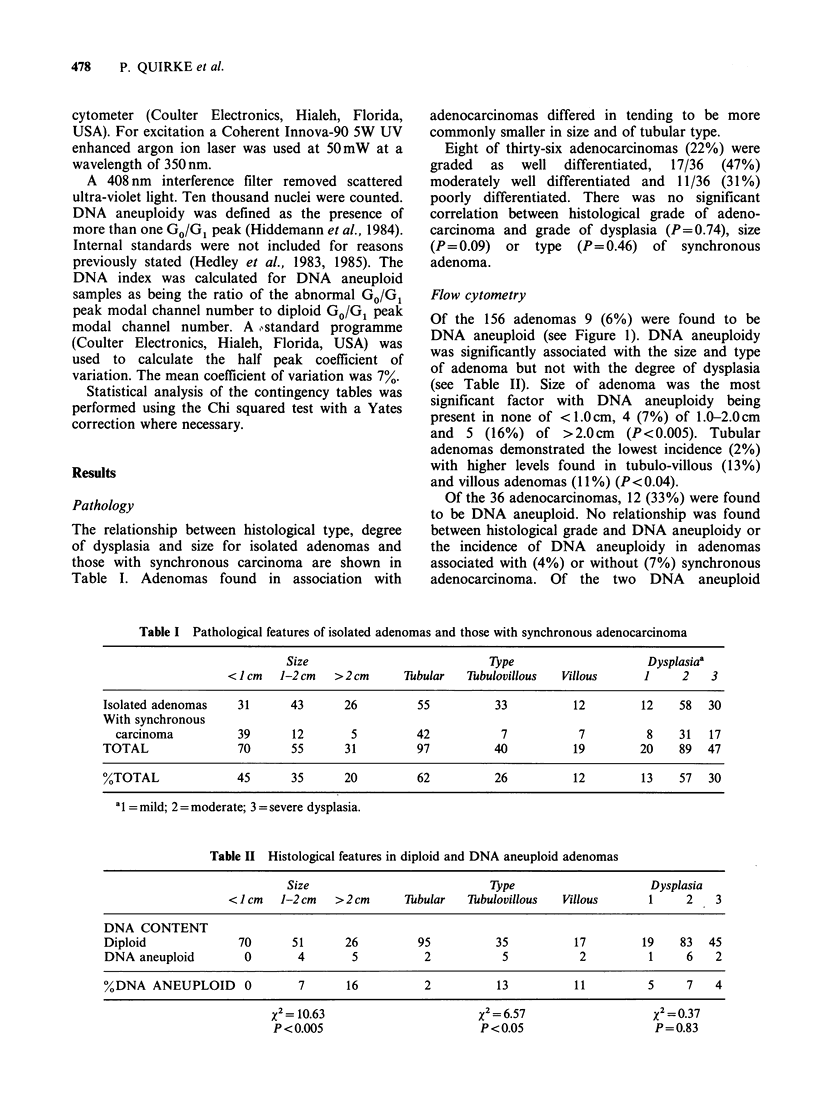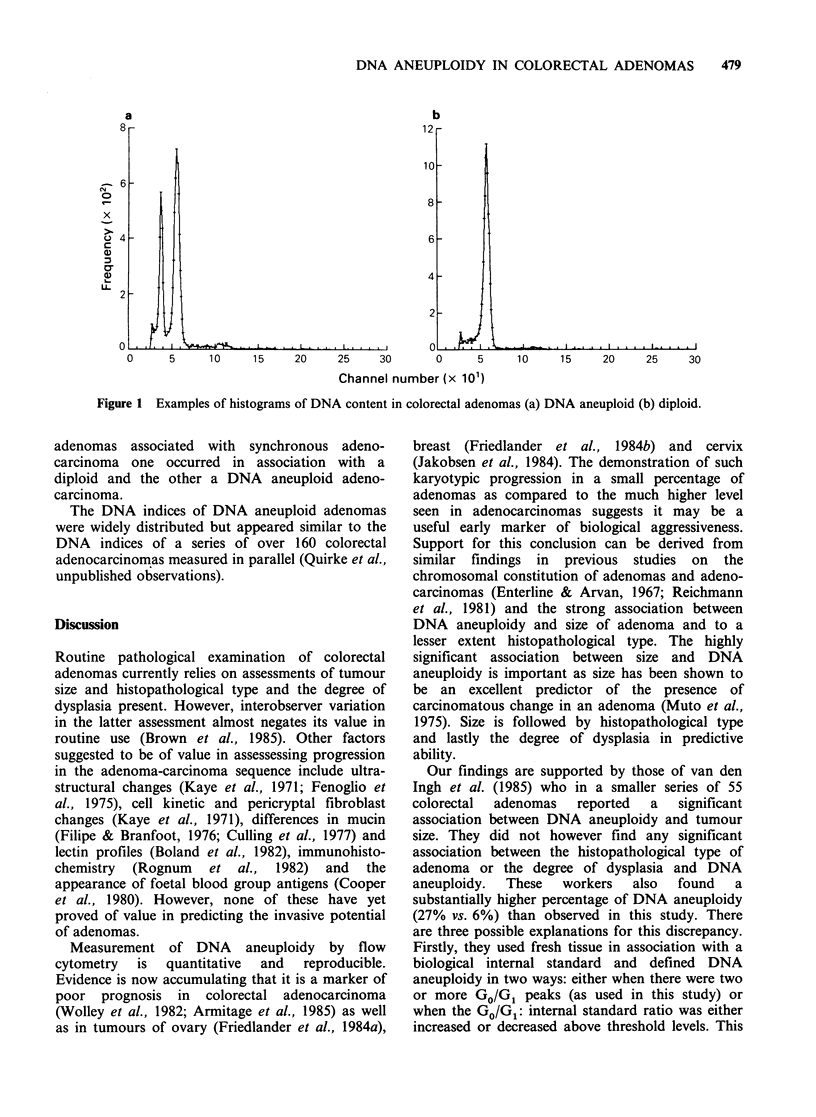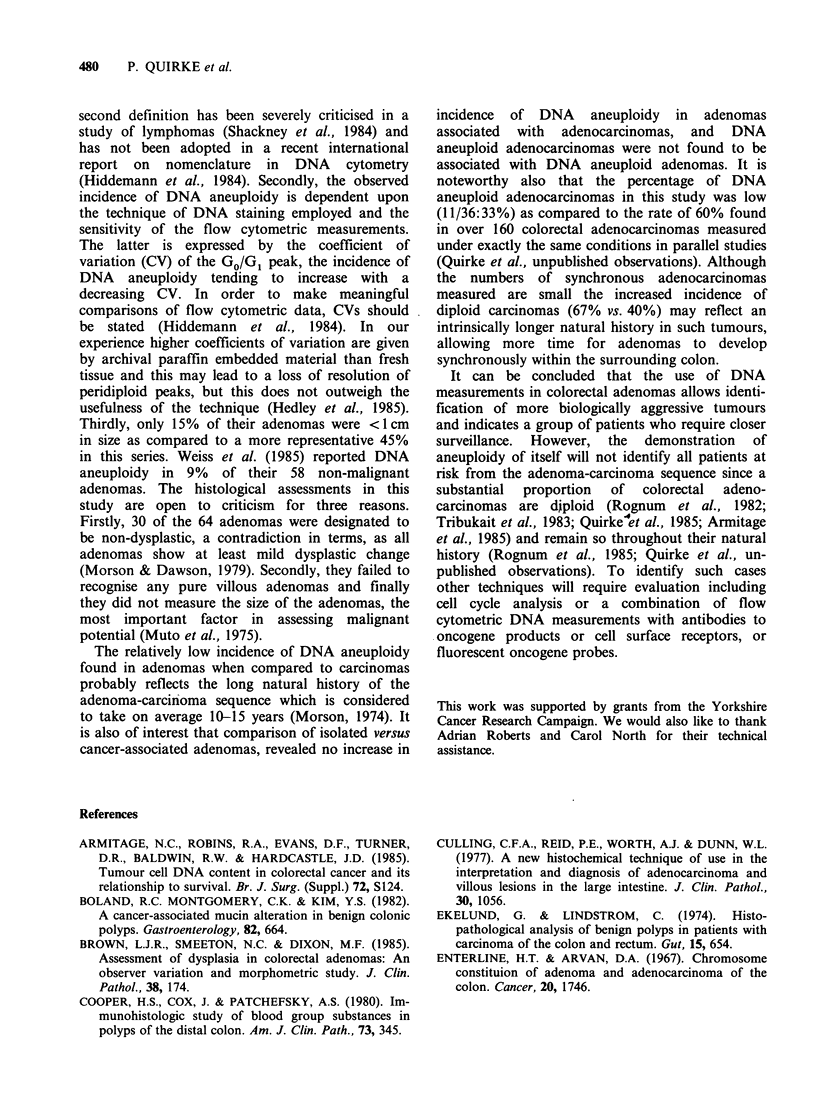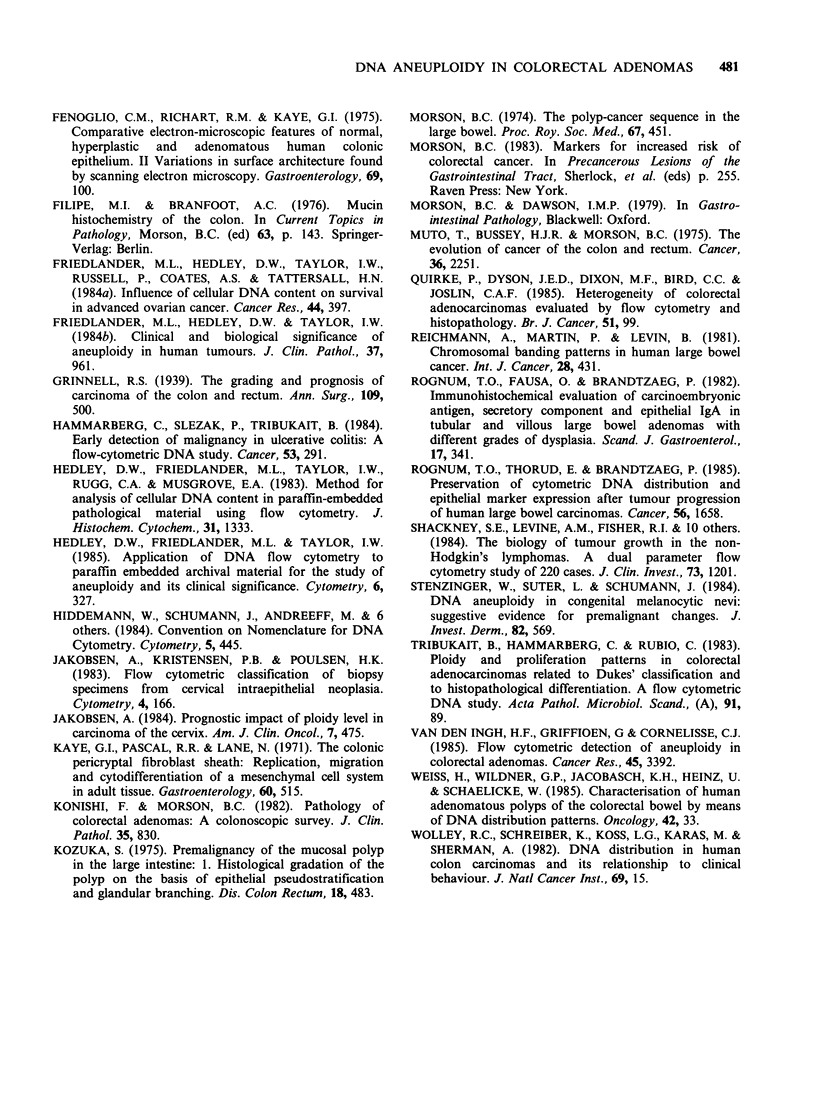# DNA aneuploidy in colorectal adenomas.

**DOI:** 10.1038/bjc.1986.75

**Published:** 1986-04

**Authors:** P. Quirke, J. B. Fozard, M. F. Dixon, J. E. Dyson, G. R. Giles, C. C. Bird

## Abstract

The frequency of DNA aneuploidy was investigated by flow cytometry in 156 colorectal adenomas including 56 associated with 36 synchronous adenocarcinomas. Nine of 156 adenomas (6%) were DNA aneuploid. DNA aneuploidy correlated with increasing size (P less than 0.005) and histopathological type P less than 0.05) but not with dysplasia. Adenomas in associated with a synchronous adenocarcinoma did not have an increased incidence of DNA aneuploidy. Adenocarcinomas found in association with adenomas tend to have a lower incidence of DNA aneuploidy then the generality of colorectal cancers.


*Br. J. Cancer* (1986), **53**, 477–481

### DNA aneuploidy in colorectal adenomas

P. Quirke[1], J.B.J. Fozard[2], M.F. Dixon[1], J.E.D. Dyson[3], G.R. Giles[2] & C.C. Bird[1]

*University Departments of Pathology[1], Surgery[2] and Radiotherapy[3], University of Leeds, Leeds LS2 9JT, UK.*

**Summary** The frequency of DNA aneuploidy was investigated by flow cytometry in 156 colorectal adenomas including 56 associated with 36 synchronous adenocarcinomas. Nine of 156 adenomas (6%) were DNA aneuploid. DNA aneuploidy correlated with increasing size ($P<0.005$) and histopathological type ($P<0.05$) but not with dysplasia. Adenomas in association with a synchronous adenocarcinoma did not have an increased incidence of DNA aneuploidy. Adenocarcinomas found in association with adenomas tend to have a lower incidence of DNA aneuploidy than the generality of colorectal cancers.

DNA aneuploidy as measured by flow cytometry is present in about 60% of colorectal adenocarcinomas (Tribukait *et al.*, 1983; Armitage *et al.*, 1985; Quirke *et al.*, unpublished observations). Early studies have suggested that aneuploidy is an important new independent prognostic variable unrelated to tumour grade or pathological stage (Wolley *et al.*, 1982; Armitage *et al.*, 1985). Studies in premalignant conditions such as long standing ulcerative colitis (Hammarberg *et al.*, 1984), cervical intraepithelial neoplasia (Jakobsen *et al.*, 1983) and giant melanocytic naevi (Stenzinger *et al.*, 1984) have shown that DNA aneuploidy can arise prior to the onset of invasive malignancy and as such may be a sensitive early indicator of biological aggressiveness. Recently, and after commencement of our own work, Van den Ingh *et al.* (1985) and Weiss *et al.* (1985) have demonstrated aneuploidy in colorectal adenomas utilising fresh tissue. However, they did not explore the relationship to synchronous carcinoma.

The study reported here was undertaken to establish the frequency of DNA aneuploidy in a large series of colorectal adenomas and to relate this to the currently accepted criteria of likely malignant transformation: severe dysplasia, histological type and size of adenoma. We have also investigated whether or not there is a higher incidence of DNA aneuploidy in adenomas found in association with synchronous adenocarcinoma.

### Materials and methods

### *Cases studied*

One hundred and fifty-six colorectal adenomas including 56 associated with 36 synchronous adenocarcinomas were retrieved from the files of the University Department of Pathology in Leeds. Haematoxylin and eosin stained (5 $\mu$m) sections of each tumour were reviewed by a single pathologist. The adenomas were graded as showing mild, moderate or severe dysplasia and divided into tubular, tubulovillous or villous type using accepted criteria (Ekelund & Lindstrom, 1974; Kozuka, 1975; and Konishi & Morson, 1982). The maximum diameter of each tumour was measured from the slide (Konishi & Morson, 1982) in order to eliminate interobserver variation in the estimation of size taken from the original gross descriptions. The adenomas were grouped according to whether they were <1 cm, 1–2 cm, or >2 cm in diameter. The colorectal carcinomas were graded into well, moderate or poor histological differentiation using accepted criteria (Grinnell, 1939).

### *Flow cytometry*

Nuclear DNA measurements were performed using a modification of the method of Hedley *et al.* (1983). Fifty $\mu$m sections were cut from paraffin embedded material and transferred to glass slides. The sections were dewaxed in xylene and rehydrated by passing through a graded series of alcohols: 100%, 95%, 90%, 70% and 50% and washing twice in distilled water. The tissue was removed from the slide with a scalpel and placed in a test tube with 0.5% pepsin (Sigma Chemical Company, Pool BH17 7NH) in 0.9% NaCl adjusted to pH 1.5 with 2N HCl, and incubated at 37°C for 30 min in a waterbath. After centrifugafugation at 2000 rpm, the pellet was washed twice in distilled water and stained by suspending in a solution ($1\,\mu g\,ml^{-1}$) of 4′-6-diamidino-2- phenylindoledihydrochloride (Boehringer Mannheim, West Germany) in RPMI 1640 tissue culture medium at 20°C for 30 min before filtering through four layers of butter muslin and syringing with a 23 gauge needle. Samples were analysed on an EPICS V flow

Correspondence: P. Quirke
Received 2 October 1985; and in revised form 11 December 1985

© The Macmillan Press Ltd., 1986

cytometer (Coulter Electronics, Hialeh, Florida, USA). For excitation a Coherent Innova-90 5W UV enhanced argon ion laser was used at 50 mW at a wavelength of 350 nm.

A 408 nm interference filter removed scattered ultra-violet light. Ten thousand nuclei were counted. DNA aneuploidy was defined as the presence of more than one $G_0/G_1$ peak (Hiddemann *et al.*, 1984). Internal standards were not included for reasons previously stated (Hedley *et al.*, 1983, 1985). The DNA index was calculated for DNA aneuploid samples as being the ratio of the abnormal $G_0/G_1$ peak modal channel number to diploid $G_0/G_1$ peak modal channel number. A standard programme (Coulter Electronics, Hialeh, Florida, USA) was used to calculate the half peak coefficient of variation. The mean coefficient of variation was 7%.

Statistical analysis of the contingency tables was performed using the Chi squared test with a Yates correction where necessary.

### Results

### *Pathology*

The relationship between histological type, degree of dysplasia and size for isolated adenomas and those with synchronous carcinoma are shown in Table I. Adenomas found in association with adenocarcinomas differed in tending to be more commonly smaller in size and of tubular type.

Eight of thirty-six adenocarcinomas (22%) were graded as well differentiated, 17/36 (47%) moderately well differentiated and 11/36 (31%) poorly differentiated. There was no significant correlation between histological grade of adenocarcinoma and grade of dysplasia ($P=0.74$), size ($P=0.09$) or type ($P=0.46$) of synchronous adenoma.

### *Flow cytometry*

Of the 156 adenomas 9 (6%) were found to be DNA aneuploid (see Figure 1). DNA aneuploidy was significantly associated with the size and type of adenoma but not with the degree of dysplasia (see Table II). Size of adenoma was the most significant factor with DNA aneuploidy being present in none of <1.0 cm, 4 (7%) of 1.0–2.0 cm and 5 (16%) of >2.0 cm ($P<0.005$). Tubular adenomas demonstrated the lowest incidence (2%) with higher levels found in tubulo-villous (13%) and villous adenomas (11%) ($P<0.04$).

Of the 36 adenocarcinomas, 12 (33%) were found to be DNA aneuploid. No relationship was found between histological grade and DNA aneuploidy or the incidence of DNA aneuploidy in adenomas associated with (4%) or without (7%) synchronous adenocarcinoma. Of the two DNA aneuploid

**Table I** Pathological features of isolated adenomas and those with synchronous adenocarcinoma

| | Size | | | Type | | | Dysplasia[a] | | |
|---|---|---|---|---|---|---|---|---|---|
| | *<1 cm* | *1–2 cm* | *>2 cm* | *Tubular* | *Tubulovillous* | *Villous* | *1* | *2* | *3* |
| Isolated adenomas | 31 | 43 | 26 | 55 | 33 | 12 | 12 | 58 | 30 |
| With synchronous carcinoma | 39 | 12 | 5 | 42 | 7 | 7 | 8 | 31 | 17 |
| TOTAL | 70 | 55 | 31 | 97 | 40 | 19 | 20 | 89 | 47 |
| %TOTAL | 45 | 35 | 20 | 62 | 26 | 12 | 13 | 57 | 30 |

[a]1 = mild; 2 = moderate; 3 = severe dysplasia.

**Table II** Histological features in diploid and DNA aneuploid adenomas

| | Size | | | Type | | | Dysplasia | | |
|---|---|---|---|---|---|---|---|---|---|
| | *<1 cm* | *1–2 cm* | *>2 cm* | *Tubular* | *Tubulovillous* | *Villous* | 1 | 2 | 3 |
| DNA CONTENT | | | | | | | | | |
| Diploid | 70 | 51 | 26 | 95 | 35 | 17 | 19 | 83 | 45 |
| DNA aneuploid | 0 | 4 | 5 | 2 | 5 | 2 | 1 | 6 | 2 |
| %DNA ANEUPLOID | 0 | 7 | 16 | 2 | 13 | 11 | 5 | 7 | 4 |
| | | $\chi^2=10.63$ $P<0.005$ | | | $\chi^2=6.57$ $P<0.05$ | | | $\chi^2=0.37$ $P=0.83$ | |

**Figure 1** Examples of histograms of DNA content in colorectal adenomas (a) DNA aneuploid (b) diploid.

adenomas associated with synchronous adenocarcinoma one occurred in association with a diploid and the other a DNA aneuploid adenocarcinoma.

The DNA indices of DNA aneuploid adenomas were widely distributed but appeared similar to the DNA indices of a series of over 160 colorectal adenocarcinomas measured in parallel (Quirke *et al.*, unpublished observations).

### Discussion

Routine pathological examination of colorectal adenomas currently relies on assessments of tumour size and histopathological type and the degree of dysplasia present. However, interobserver variation in the latter assessment almost negates its value in routine use (Brown *et al.*, 1985). Other factors suggested to be of value in assessessing progression in the adenoma-carcinoma sequence include ultrastructural changes (Kaye *et al.*, 1971; Fenoglio *et al.*, 1975), cell kinetic and pericryptal fibroblast changes (Kaye *et al.*, 1971), differences in mucin (Filipe & Branfoot, 1976; Culling *et al.*, 1977) and lectin profiles (Boland *et al.*, 1982), immunohistochemistry (Rognum *et al.*, 1982) and the appearance of foetal blood group antigens (Cooper *et al.*, 1980). However, none of these have yet proved of value in predicting the invasive potential of adenomas.

Measurement of DNA aneuploidy by flow cytometry is quantitative and reproducible. Evidence is now accumulating that it is a marker of poor prognosis in colorectal adenocarcinoma (Wolley *et al.*, 1982; Armitage *et al.*, 1985) as well as in tumours of ovary (Friedlander *et al.*, 1984*a*), breast (Friedlander *et al.*, 1984*b*) and cervix (Jakobsen *et al.*, 1984). The demonstration of such karyotypic progression in a small percentage of adenomas as compared to the much higher level seen in adenocarcinomas suggests it may be a useful early marker of biological aggressiveness. Support for this conclusion can be derived from similar findings in previous studies on the chromosomal constitution of adenomas and adenocarcinomas (Enterline & Arvan, 1967; Reichmann *et al.*, 1981) and the strong association between DNA aneuploidy and size of adenoma and to a lesser extent histopathological type. The highly significant association between size and DNA aneuploidy is important as size has been shown to be an excellent predictor of the presence of carcinomatous change in an adenoma (Muto *et al.*, 1975). Size is followed by histopathological type and lastly the degree of dysplasia in predictive ability.

Our findings are supported by those of van den Ingh *et al.* (1985) who in a smaller series of 55 colorectal adenomas reported a significant association between DNA aneuploidy and tumour size. They did not however find any significant association between the histopathological type of adenoma or the degree of dysplasia and DNA aneuploidy. These workers also found a substantially higher percentage of DNA aneuploidy (27% *vs.* 6%) than observed in this study. There are three possible explanations for this discrepancy. Firstly, they used fresh tissue in association with a biological internal standard and defined DNA aneuploidy in two ways: either when there were two or more $G_0/G_1$ peaks (as used in this study) or when the $G_0/G_1$: internal standard ratio was either increased or decreased above threshold levels. This second definition has been severely criticised in a study of lymphomas (Shackney *et al.*, 1984) and has not been adopted in a recent international report on nomenclature in DNA cytometry (Hiddemann *et al.*, 1984). Secondly, the observed incidence of DNA aneuploidy is dependent upon the technique of DNA staining employed and the sensitivity of the flow cytometric measurements. The latter is expressed by the coefficient of variation (CV) of the $G_0/G_1$ peak, the incidence of DNA aneuploidy tending to increase with a decreasing CV. In order to make meaningful comparisons of flow cytometric data, CVs should be stated (Hiddemann *et al.*, 1984). In our experience higher coefficients of variation are given by archival paraffin embedded material than fresh tissue and this may lead to a loss of resolution of peridiploid peaks, but this does not outweigh the usefulness of the technique (Hedley *et al.*, 1985). Thirdly, only 15% of their adenomas were <1 cm in size as compared to a more representative 45% in this series. Weiss *et al.* (1985) reported DNA aneuploidy in 9% of their 58 non-malignant adenomas. The histological assessments in this study are open to criticism for three reasons. Firstly, 30 of the 64 adenomas were designated to be non-dysplastic, a contradiction in terms, as all adenomas show at least mild dysplastic change (Morson & Dawson, 1979). Secondly, they failed to recognise any pure villous adenomas and finally they did not measure the size of the adenomas, the most important factor in assessing malignant potential (Muto *et al.*, 1975).

The relatively low incidence of DNA aneuploidy found in adenomas when compared to carcinomas probably reflects the long natural history of the adenoma-carcinoma sequence which is considered to take on average 10–15 years (Morson, 1974). It is also of interest that comparison of isolated *versus* cancer-associated adenomas, revealed no increase in incidence of DNA aneuploidy in adenomas associated with adenocarcinomas, and DNA aneuploid adenocarcinomas were not found to be associated with DNA aneuploid adenomas. It is noteworthy also that the percentage of DNA aneuploid adenocarcinomas in this study was low (11/36:33%) as compared to the rate of 60% found in over 160 colorectal adenocarcinomas measured under exactly the same conditions in parallel studies (Quirke *et al.*, unpublished observations). Although the numbers of synchronous adenocarcinomas measured are small the increased incidence of diploid carcinomas (67% *vs.* 40%) may reflect an intrinsically longer natural history in such tumours, allowing more time for adenomas to develop synchronously within the surrounding colon.

It can be concluded that the use of DNA measurements in colorectal adenomas allows identification of more biologically aggressive tumours and indicates a group of patients who require closer surveillance. However, the demonstration of aneuploidy of itself will not identify all patients at risk from the adenoma-carcinoma sequence since a substantial proportion of colorectal adenocarcinomas are diploid (Rognum *et al.*, 1982; Tribukait *et al.*, 1983; Quirke *et al.*, 1985; Armitage *et al.*, 1985) and remain so throughout their natural history (Rognum *et al.*, 1985; Quirke *et al.*, unpublished observations). To identify such cases other techniques will require evaluation including cell cycle analysis or a combination of flow cytometric DNA measurements with antibodies to oncogene products or cell surface receptors, or fluorescent oncogene probes.

This work was supported by grants from the Yorkshire Cancer Research Campaign. We would also like to thank Adrian Roberts and Carol North for their technical assistance.

### References

ARMITAGE, N.C., ROBINS, R.A., EVANS, D.F., TURNER, D.R., BALDWIN, R.W. & HARDCASTLE, J.D. (1985). Tumour cell DNA content in colorectal cancer and its relationship to survival. *Br. J. Surg.* (Suppl.) **72,** S124.

BOLAND, R.C. MONTGOMERY, C.K. & KIM, Y.S. (1982). A cancer-associated mucin alteration in benign colonic polyps. *Gastroenterology,* **82,** 664.

BROWN, L.J.R., SMEETON, N.C. & DIXON, M.F. (1985). Assessment of dysplasia in colorectal adenomas: An observer variation and morphometric study. *J. Clin. Pathol.,* **38,** 174.

COOPER, H.S., COX, J. & PATCHEFSKY, A.S. (1980). Immunohistologic study of blood group substances in polyps of the distal colon. *Am. J. Clin. Path.,* **73,** 345.

CULLING, C.F.A., REID, P.E., WORTH, A.J. & DUNN, W.L. (1977). A new histochemical technique of use in the interpretation and diagnosis of adenocarcinoma and villous lesions in the large intestine. *J. Clin. Pathol.,* **30,** 1056.

EKELUND, G. & LINDSTROM, C. (1974). Histopathological analysis of benign polyps in patients with carcinoma of the colon and rectum. *Gut,* **15,** 654.

ENTERLINE, H.T. & ARVAN, D.A. (1967). Chromosome constituion of adenoma and adenocarcinoma of the colon. *Cancer,* **20,** 1746.

FENOGLIO, C.M., RICHART, R.M. & KAYE, G.I. (1975). Comparative electron-microscopic features of normal, hyperplastic and adenomatous human colonic epithelium. II Variations in surface architecture found by scanning electron microscopy. *Gastroenterology*, **69**, 100.

FILIPE, M.I. & BRANFOOT, A.C. (1976). Mucin histochemistry of the colon. In *Current Topics in Pathology*, Morson, B.C. (ed) **63**, p. 143. Springer-Verlag: Berlin.

FRIEDLANDER, M.L., HEDLEY, D.W., TAYLOR, I.W., RUSSELL, P., COATES, A.S. & TATTERSALL, H.N. (1984*a*). Influence of cellular DNA content on survival in advanced ovarian cancer. *Cancer Res.*, **44**, 397.

FRIEDLANDER, M.L., HEDLEY, D.W. & TAYLOR, I.W. (1984*b*). Clinical and biological significance of aneuploidy in human tumours. *J. Clin. Pathol.*, **37**, 961.

GRINNELL, R.S. (1939). The grading and prognosis of carcinoma of the colon and rectum. *Ann. Surg.*, **109**, 500.

HAMMARBERG, C., SLEZAK, P., TRIBUKAIT, B. (1984). Early detection of malignancy in ulcerative colitis: A flow-cytometric DNA study. *Cancer*, **53**, 291.

HEDLEY, D.W., FRIEDLANDER, M.L., TAYLOR, I.W., RUGG, C.A. & MUSGROVE, E.A. (1983). Method for analysis of cellular DNA content in paraffin-embedded pathological material using flow cytometry. *J. Histochem. Cytochem.*, **31**, 1333.

HEDLEY, D.W., FRIEDLANDER, M.L. & TAYLOR, I.W (1985). Application of DNA flow cytometry to paraffin embedded archival material for the study of aneuploidy and its clinical significance. *Cytometry*, **6**, 327.

HIDDEMANN, W., SCHUMANN, J., ANDREEFF, M. & 6 others. (1984). Convention on Nomenclature for DNA Cytometry. *Cytometry*, **5**, 445.

JAKOBSEN, A., KRISTENSEN, P.B. & POULSEN, H.K. (1983). Flow cytometric classification of biopsy specimens from cervical intraepithelial neoplasia. *Cytometry*, **4**, 166.

JAKOBSEN, A. (1984). Prognostic impact of ploidy level in carcinoma of the cervix. *Am. J. Clin. Oncol.*, **7**, 475.

KAYE, G.I., PASCAL, R.R. & LANE, N. (1971). The colonic pericryptal fibroblast sheath: Replication, migration and cytodifferentiation of a mesenchymal cell system in adult tissue. *Gastroenterology*, **60**, 515.

KONISHI, F. & MORSON, B.C (1982). Pathology of colorectal adenomas: A colonoscopic survey. *J. Clin. Pathol.* **35**, 830.

KOZUKA, S. (1975). Premalignancy of the mucosal polyp in the large intestine: 1. Histological gradation of the polyp on the basis of epithelial pseudostratification and glandular branching. *Dis. Colon Rectum*, **18**, 483.

MORSON, B.C. (1974). The polyp-cancer sequence in the large bowel. *Proc. Roy. Soc. Med.*, **67**, 451.

MORSON, B.C. (1983). Markers for increased risk of colorectal cancer. In *Precancerous Lesions of the Gastrointestinal Tract*, Sherlock, *et al.* (eds) p. 255. Raven Press: New York.

MORSON, B.C. & DAWSON, I.M.P. (1979). In *Gastrointestinal Pathology*, Blackwell: Oxford.

MUTO, T., BUSSEY, H.J.R. & MORSON, B.C. (1975). The evolution of cancer of the colon and rectum. *Cancer*, **36**, 2251.

QUIRKE, P., DYSON, J.E.D., DIXON, M.F., BIRD, C.C. & JOSLIN, C.A.F. (1985). Heterogeneity of colorectal adenocarcinomas evaluated by flow cytometry and histopathology. *Br. J. Cancer*, **51**, 99.

REICHMANN, A., MARTIN, P. & LEVIN, B. (1981). Chromosomal banding patterns in human large bowel cancer. *Int. J. Cancer*, **28**, 431.

ROGNUM, T.O., FAUSA, O. & BRANDTZAEG, P. (1982). Immunohistochemical evaluation of carcinoembryonic antigen, secretory component and epithelial IgA in tubular and villous large bowel adenomas with different grades of dysplasia. *Scand. J. Gastroenterol.*, **17**, 341.

ROGNUM, T.O., THORUD, E. & BRANDTZAEG, P. (1985). Preservation of cytometric DNA distribution and epithelial marker expression after tumour progression of human large bowel carcinomas. *Cancer*, **56**, 1658.

SHACKNEY, S.E., LEVINE, A.M., FISHER, R.I. & 10 others. (1984). The biology of tumour growth in the non-Hodgkin's lymphomas. A dual parameter flow cytometry study of 220 cases. *J. Clin. Invest.*, **73**, 1201.

STENZINGER, W., SUTER, L. & SCHUMANN, J. (1984). DNA aneuploidy in congenital melanocytic nevi: suggestive evidence for premalignant changes. *J. Invest. Derm.*, **82**, 569.

TRIBUKAIT, B., HAMMARBERG, C. & RUBIO, C. (1983). Ploidy and proliferation patterns in colorectal adenocarcinomas related to Dukes' classification and to histopathological differentiation. A flow cytometric DNA study. *Acta Pathol. Microbiol. Scand.*, (A), **91**, 89.

VAN DEN INGH, H.F., GRIFFIOEN, G & CORNELISSE, C.J. (1985). Flow cytometric detection of aneuploidy in colorectal adenomas. *Cancer Res.*, **45**, 3392.

WEISS, H., WILDNER, G.P., JACOBASCH, K.H., HEINZ, U. & SCHAELICKE, W. (1985). Characterisation of human adenomatous polyps of the colorectal bowel by means of DNA distribution patterns. *Oncology*, **42**, 33.

WOLLEY, R.C., SCHREIBER, K., KOSS, L.G., KARAS, M. & SHERMAN, A. (1982). DNA distribution in human colon carcinomas and its relationship to clinical behaviour. *J. Natl Cancer Inst.*, **69**, 15.